# Zwitterionic Cellulose Hydrogels for Flexible Strain Sensors with Enhanced Sensing and Mechanical Performance

**DOI:** 10.1021/acsapm.5c03412

**Published:** 2025-10-23

**Authors:** Maryam Madani, Sedigheh Borandeh, Hossein Baniasadi, Fevzihan Basarir, Jaana Vapaavuori, Jukka Seppälä, Jukka Niskanen

**Affiliations:** † Polymer Synthesis Technology, Department of Chemical and Metallurgical Engineering, School of Chemical Engineering, 154262 Aalto University, P.O. Box 16100, FI-00076 Espoo, Finland; ‡ Department of Chemistry and Materials Science, Aalto University, P.O. Box 16100, FI-00076 Espoo, Finland

**Keywords:** Ion conductive hydrogel, Polyzwitterion, Cellulose, Strain sensor, Antifreezing, Self-adhesion

## Abstract

Conductive hydrogels combine flexibility, conductivity, and adaptability, making them ideal for flexible strain sensors. However, achieving multifunctional performance under freezing conditions remains challenging, as flexibility, adhesion, and conductivity often deteriorate at high or low temperatures. In this work, we introduce a polyzwitterion-hydroxyethylcellulose (HEC) hydrogel that transforms into a freeze-resistant ion-conducting material. The mechanical properties and ion conductivity of this hydrogel are enhanced through an optimized composition, with HEC’s structure playing a crucial role in its performance. The interconnected network, fortified by intermolecular forces and charged polar end groups, delivers exceptional properties, including a tensile strength of 54 kPa, a gauge factor of 1.63, and a response time of 2.11 s. These characteristics enable the hydrogel sensor to accurately monitor human motion, establishing an ideal platform for iontronics, soft robotics, and advanced health diagnostics.

## Graphical abstract

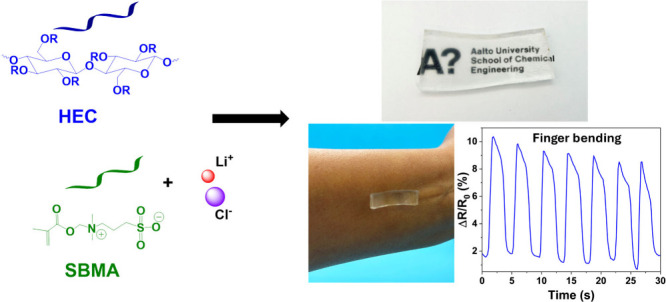

## 1 Introduction

The rapid advancement of wearable technology has sparked significant interest in the development of advanced materials that not only enhance the functionality and flexibility of wearable devices but also ensure biocompatibility.^1–3^ Among the diverse range of materials explored, ion-conductive hydrogels have emerged as promising candidates. These hydrogels stand out due to their exceptional mechanical properties, inherent flexibility, and ability to interface seamlessly with biological tissues, making them ideal for applications in health monitoring and soft robotics.^4,5^ Their ability to convert mechanical forces into electrical signals—manifested as a change in resistance, current, or capacitance—opens new possibilities for precise, real-time sensing and signal transduction in wearable devices.^6,7^

Efficient wearable sensors require materials that are not only highly sensitive and conductive but also flexible, durable, and biocompatible. Hydroxyethylcellulose (HEC) is a nonionic and water-soluble cellulose derivative.^8,9^ It is widely used in pharmaceuticals, cosmetics, and food applications due to its biocompatibility and excellent film-forming properties.^10–12^ However, its utilization in wearable sensors has been limited due to its low conductivity. The properties of HEC can be modified by grafting polymers to extend its range of applications from the hydroxyl groups while maintaining its biocompatibility and flexibility.^13^ HEC has been grafted with acrylic acid and acrylamide by radical polymerization to prepare superabsorbents and for drug delivery of diclofenac.^14,15^

Polyzwitterions contain both positive and negative charges within the same repeating unit, which gives it a high dipole moment and numerous charged groups that make them ion-conducting.^16,17^ Although zwitterionic polymers maintain an overall electrically neutral molecular chain, the coexistence of oppositely charged groups results in high polarity and excellent hydrophilicity. Additionally, the polymer’s electrical properties can be further tuned by environmental factors such as pH and salt ions, significantly expanding its range of applications.^18,19^ For instance, sulfobetaine methacrylate (2-(methacryloyloxy)­ethyl dimethyl-(3-sulfopropyl) ammonium hydroxide, SB) contains an anionic sulfonic group and a quaternary ammonium group as the cationic group, which can facilitate ion transport of added ions through ionic interactions.^20,21^ Zwitterionic polymers are also known for their self-adhesive properties, which are crucial for wearable devices due to the dipole–dipole and electrostatic interactions between the hydrogel and the substrate. Hao et al. reported an adhesive zwitterionic hydrogel for strain and temperature sensors, prepared from TEMPO-oxidized cellulose-polyaniline nanofibers incorporated in a sulfobetaine methacrylate and acrylamide copolymer matrix. These hydrogels were unique in their ability to combine excellent flexibility, self-adhesion, conductivity, and thermal sensitivity.^22^

This study explores the synthesis of hydrogels by grafting polyzwitterions onto hydroxyethylcellulose (HEC) through a one-pot method, combining the advantageous properties of both components. The fabrication process involves the free radical polymerization of sulfobetaine methacrylate with the cross-linker N,N′-methylenebis­(acrylamide), in the presence of HEC and lithium chloride (LiCl) in an aqueous medium. We hypothesize that grafting polyzwitterions onto HEC will enhance the mechanical, ionic, and thermal properties of the resulting hydrogels, making them more suitable for wearable, flexible sensors. Specifically, we anticipate that the polyzwitterion integration will improve low-temperature tolerance, tensile strength, adhesion, and transparency, addressing the limitations of conventional hydrogel-based sensors. These enhanced properties position the hydrogels as viable candidates for next-generation wearable sensors that can withstand mechanical stresses while maintaining functionality in diverse environments. Furthermore, their transparency and adhesion capabilities offer potential applications in optical sensing and skin-mounted devices, broadening their utility beyond traditional sensor technologies.

## 2 Experimental Section

### 2.1 Materials

Hydroxyethylcellulose (HEC, *M*_w_ = 250 000 g/mol), 2-(methacryloyloxy)­ethyl dimethyl-(3-sulfopropyl) ammonium hydroxide (sulfobetaine methacrylate, SB, 95%), ammonium persulfate (APS, 98%) and *N*,*N*,*N*′,*N*′-tetramethylethylenediamine (TEMED, 99%) were purchased from Sigma-Aldrich and used as received. *N*,*N*′-Methylenebis­(acrylamide) (MBAA, > 98%), and lithium chloride (LiCl, > 98%) were obtained from Tokyo Chemical Industry Co (TCI).

### 2.2 Fabrication of Polysulfobetaine-Hydroxyethylcellulose Hydrogels

A simple one-pot method was used to prepare the polysulfobetaine-hydroxyethylcellulose (PSB-HEC) hydrogels. First, a certain amount of HEC was dissolved in 8 mL of deionized water in percentage ratios of 1, 3, and 5 according to the SB weight. In the next step, 0.5 g (11.7 mmol) of LiCl was added to the solution, yielding a 1.5 M LiCl solution, and stirred for 10 min. Then, 2.3 g (8.23 mmol) of SB, 35 mg (0.15 mmol) of APS, 10 μL (7.76 mg, 0.06 mmol) TEMED, and 11.5 mg (0.08 mmol) of MBAA cross-linker were added to the solution and stirred for 5 min at room temperature. The solution was then poured into a silicon mold, and the hydrogel was formed within 24 h via radical polymerization. PSB-HEC hydrogels with varying amounts of HEC were prepared as follows: 0, 1.0, 3.0, and 5.0 wt % and were designated as PSB-HEC, PSB-HEC1, PSB-HEC3, and PSB-HEC5. Among these, PSB-HEC3 was selected to be studied further, as it represents an intermediate HEC content, providing a balanced combination of mechanical strength, flexibility, and ionic conductivity. The relevant explanation has been added to the revised manuscript.

### 2.3 Characterizations

#### 2.3.1 Fourier Transform Infrared Spectroscopy

The chemical structure of freeze-dried hydrogels was characterized by Fourier transforms infrared (FTIR) spectroscopy. All components were recorded by an attenuated total reflectance (ATR–IR) PerkinElmer spectrometer in transmission mode, with a scan range of 500–4000 cm^–1^, with a resolution of 4 cm^–1^, and 16 accumulations.

#### 2.3.2 Optical Transparency Measurement

The transmittance of hydrogel PSB-HEC3 (3 mm thick) was measured across a wavelength range of 200 to 800 nm using a VWR UV-3100PC spectrophotometer. The hydrogel sample was prepared and placed in the spectrophotometer’s light path to assess its optical transparency. The transmittance data were recorded to evaluate the hydrogel’s ability to transmit light. Each sample was tested at least three times, and the results are reported as the average value ± standard deviation.

#### 2.3.3 Rheology Measurements

The viscoelastic properties of the hydrogels were measured using a rheometer (Anton Paar MCR 301, Austria). Hydrogel disks were made using a silicon mold with a diameter of 25 mm, resulting in disks with a thickness of 3 mm. First, dynamic strain measurements were conducted with a range of 0.01–100% strain at a fixed frequency of 10 rad s^–1^ to find the linear viscoelastic region. After that, a frequency sweep test was performed using a PP25 geometry at room temperature from 0.1 to 100 rad s^–1^ with the linear viscoelastic region, e.g., a fixed strain of 1%. All measurements were done at ambient temperature (23 °C).

#### 2.3.4 Mechanical Testing

The mechanical characteristics of the hydrogels were investigated using a Universal Tester, Instron model 4204. The hydrogel specimens (2.7 mm × 60 mm) were stretched at a speed of 20 mm/min with a load cell of 1 kN. For compression studies, specimens with a diameter of 10 mm and a thickness of 15 mm were used. The sample was compressed up to 85% of its initial height with a compression speed of 20 mm/min. Cyclic loading–unloading compression experiments were conducted at 100% strain over 5 cycles. A stress–strain curve was integrated to calculate the fracture toughness of the hydrogel. Each sample was tested at least three times, and the results are reported as the average value ± standard deviation.

#### 2.3.5 Adhesion Testing

The hydrogel samples were first applied to the surfaces of different substrates, covering a bonded area with a diameter of 10 mm. The samples were then hung upside down for visual inspection and photographed. To evaluate adhesion performance, a Universal Tester (Instron Model 4204) was employed. Hydrogel specimens (10 mm × 10 mm) were sandwiched between two paper sheets (10 mm × 30 mm) to create a bonded area. The paper sheets were subjected to a shear rate of 20 mm/min at room temperature until separation occurred, allowing for the measurement of the hydrogels’ adhesion strength. Each sample was tested at least three times, and the results are reported as the average value ± standard deviation.

#### 2.3.6 Antifreezing Testing

Hydrogels were tested for freezing resistance by cutting them into small pieces and using a differential scanning calorimeter (TA Instruments Discovery DSC). The heating/cooling rate was set at 10 °C min^–1^ between −50 to 50 °C under a nitrogen atmosphere. Each sample was tested through two consecutive heating–cooling cycles, and the representative data from the second cycle are reported to ensure reproducibility and stability of the thermal response.

#### 2.3.7 Water Content Measurement

The water content of the hydrogel is determined by gravimetric analysis. After the hydrogel is formed, a sample is weighed in its fully swollen state (W_s_), then placed in an oven at 105 °C and dried until it reaches a constant weight (W_d_). The water content is calculated using the following formula:

1 $$Water\;content\;\left(\%\right)=\frac{W_{s}-W_{d}}{W_{s}}\times 100$$

Each sample was tested at least three times, and the results are reported as the average value ± standard deviation.

#### 2.3.8 Sensing Performance

Hydrogel electrical conductivity was determined by using a digital multimeter at room temperature, using the following equation:

2 σ=L/(R×S)

where σ is the ionic conductivity (S/m), L is the sample thickness (m), R is the resistance of the composite hydrogel, and S is the cross-sectional area of the hydrogel sample.

Using a KEYSIGHT LCR meter (E4980AL), sensor resistance values were measured at a frequency of 10 kHz using an AC voltage of 1 V. The sensors were subjected to external tensile stress using a mechanical analyzer equipped with a computer-controlled stage (TA.XTplus, Stable Micro Systems). The resistance change was calculated according to the following equation:

$$\frac{\Delta R}{R_{0}}\;\left(\%\right)=\frac{\left(R_{t}-R_{0}\right)}{R_{0}}\times 100$$ 3

where R_0_ and R_t_ are the electric resistance without and with applied strain, respectively.

The gauge factor (GF) of the hydrogel sensor was defined using eq 4.

4 $$GF=\frac{\left(\Delta R/R_{0}\right)}{\varepsilon }$$

where ΔR = R_t_ – R_0_ and ε is the strain of the hydrogel, respectively.

Detection of human motions (elbow, finger, wrist, and knee movements) was carried out by placing the PSB-HEC3 at designated points on the human. Written consent from all participants was obtained prior to the research (Aalto University Research Ethics Committee with D/1302/03.04/2022 WEARSENSNANO decision number)

#### 2.3.9 Resistance Measurement and Sensor Performance Analysis

For the resistance measurements, three identical samples were used, and the data is presented as an average with a standard deviation. The sensor performance tests were performed on one sample. The movement detection characterizations of one sample were performed. MATLAB and MS Excel were used to analyze the data. Each sample was tested at least three times, and the results are reported as the average value ± standard deviation.

## 3 Results and Discussion

### 3.1 Fabrication Strategy and Characterization of PSB-HEC Hydrogels

The hydrogels were prepared by polymerization and cross-linking of SB with MBAA in an aqueous solution containing HEC and LiCl. Polymerization was initiated by a redox initiator system formed by persulfate and TEMED, which facilitates the grafting of cellulosic materials with polymers via radical polymerization.^23^ After 24 h at room temperature, a hydrogel was obtained, cross-linked through both chemical and physical bonds. Chemical cross-links were formed by MBAA, while physical cross-links resulted from intra- and interchain electrostatic interactions and hydrogen bonding between the PSB grafts and HEC. It should be noted that PSB is a UCST-type thermoresponsive polymer.^24–26^ However, due to the presence of LiCl and the physical cross-links present, no thermoresponsiveness of the gels was observed while cooling the gels to 0 °C. Hydrogels composed solely of PSB are known to be fragile, which is why the inclusion of HEC is necessary for reinforcement.^27^ Due to the presence of LiCl in the preparation solution, the hydrogel becomes interpenetrated with lithium and chloride ions. The water content of these hydrogels was determined to be 27 wt %. It is important to note that in the presence of LiCl (1.5 M), electrostatic interactions between the zwitterionic moieties of PSB are screened by the ions, as are the hydrogen bonds between the carbonyl groups of PSB and HEC.^25,28,29^ The association of ions with the zwitterionic groups is advantageous for the conductivity of the hydrogel, as these ions facilitate the migration of lithium ions through ion hopping.^27,30,31^ A schematic representation of the PSB-HEC hydrogels and their transparency is shown in Figure 1. The high transparency of the PSB-HEC hydrogels allows for clear visibility of underlying objects.

**Figure 1:**
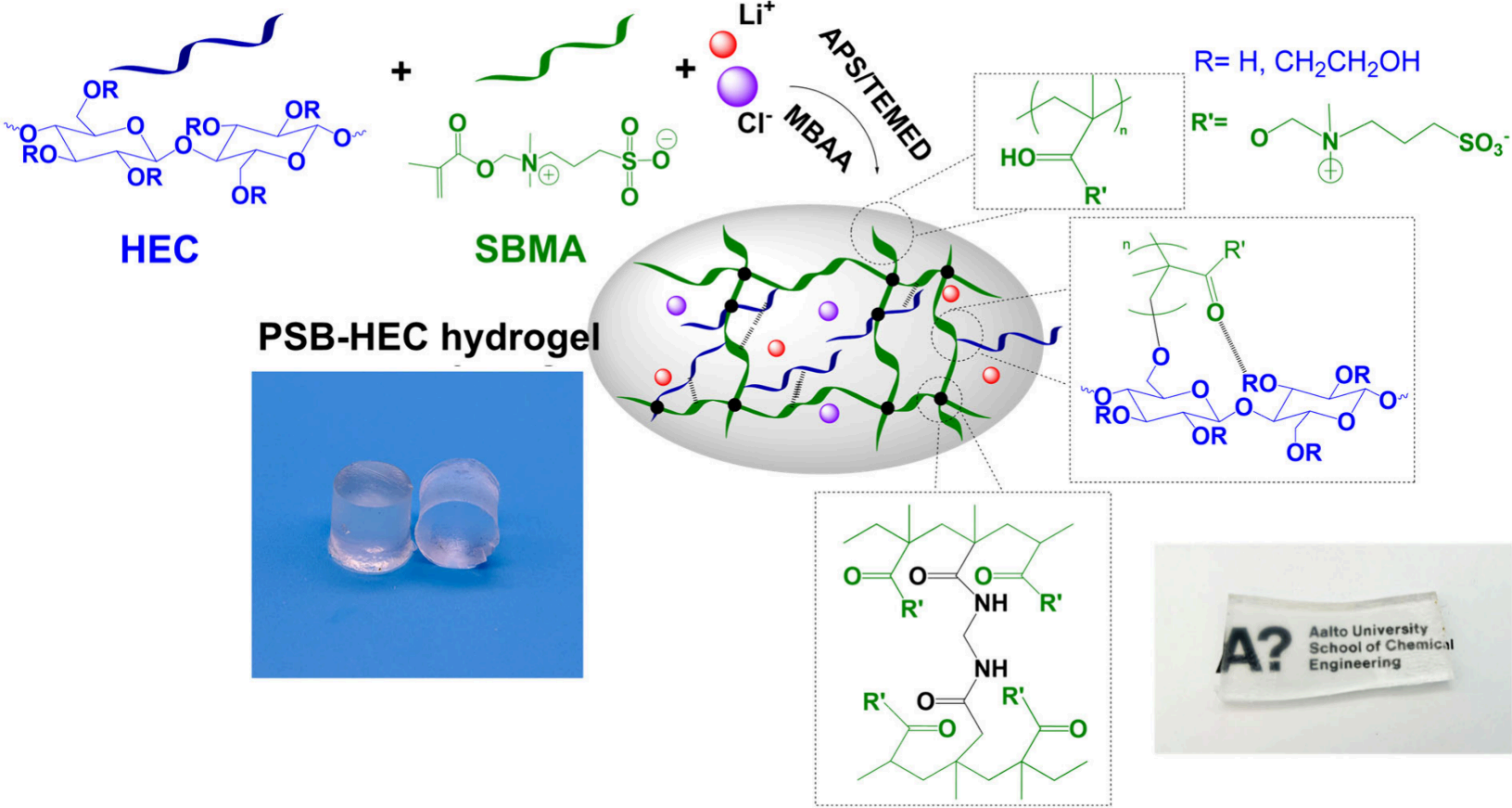
Schematic illustration of the synthesis of PSB-HEC3 with photographs of the transparent hydrogel.

The FTIR spectra of HEC, PSB, and PSB-HEC3 are presented in Figure 2a. The FTIR spectra show that the characteristic stretching vibration band of hydroxyl groups at 3379 cm^–1^ of HEC became weaker after the hydrogel formation in PSB-HEC3. Furthermore, two distinct bands appeared at 2842 and 2910 cm^–1^ in the hydrogel sample PSB-HEC3. These bands are assigned to the methylene and methyl groups from the cross-linked PSB. The carbonyl and sulfonyl groups of the PSB are observed from the bands at 1725, 1041, and 1001 cm^–1^.^32^ It should be noted that the band for the characteristic stretching vibration of the double bonds (C = C) at 1610 cm^–1^ is not observed after the polymerization. The disappearance of the C = C absorbance band indicates that the monomers have been consumed during the polymerization process, resulting in the formation of cross-linked polymer chains.^32^ Furthemore, the two peaks around 1500 cm^–1^ in the FTIR spectrum of HEC are associated with CH_2_ bending and cellulose skeletal vibrations—often considered markers of crystallinity. After polymerization in PSB-HEC3, these bands are no longer evident. Similar observations have been reported in the literature, where cross-linking and hydrogel formation led to decreased intensity or disappearance of these peaks due to reduced crystallinity and overlapping signals from newly formed polymer networks.^33,34^

**Figure 2:**
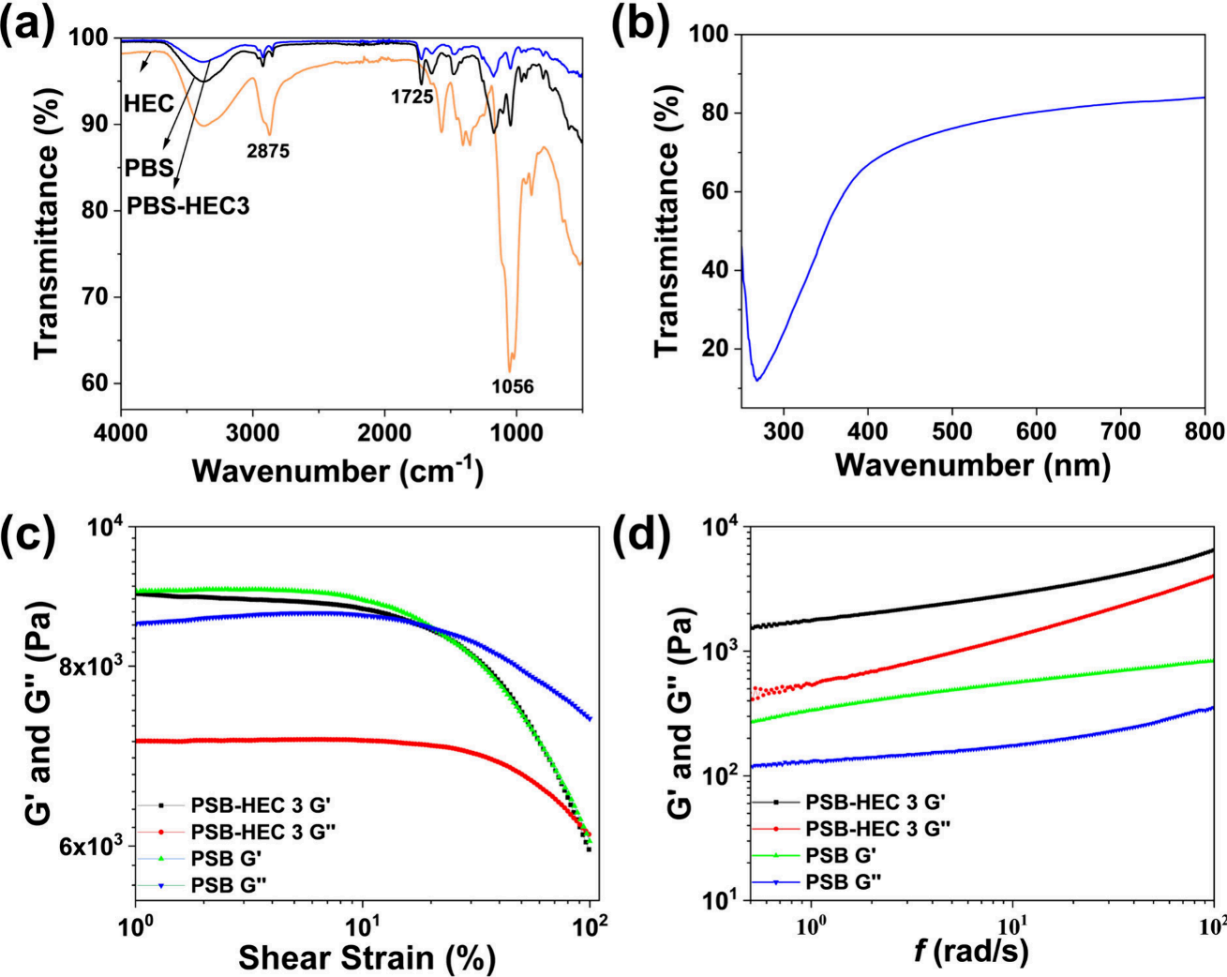
(a) FTIR spectra of HEC, PSB, and PSB-HEC3, (b) UV-transmittance of PSB-HEC3 hydrogel, (c) strain dependence, and (d) frequency sweep test for PSB and PSB-HEC3 hydrogels.

As shown in Figure 2b, the PSB-HEC3 hydrogel exhibits 84% transmittance at 600 nm, a standard reference point for evaluating optical transparency, demonstrating its excellent clarity across the visible light range (400–800 nm). This high level of transmittance is advantageous for wearable devices and sensors that require unobstructed visibility and integration with the human body or surrounding environments, ensuring that the hydrogel can be used effectively in applications such as flexible strain sensors, where clear visibility of the underlying surface is essential for both aesthetic and functional purposes. However, the transmittance of the hydrogel significantly decreases in the UV region from 400 to 250 nm, reaching a minimum around 250 nm before gradually increasing at shorter wavelengths. This selective UV blocking is attributed to the presence of charged sulfonate and quaternary ammonium groups in the sulfobetaine monomer, which absorb UV light through electronic transitions, preventing UV transmission.^35,36^ The absorption of UV light could be beneficial in specific applications where UV shielding or light filtering is required. The hydrogel’s capacity to block UV radiation can protect sensors used in wearable devices.^37^ The combination of high visible transmittance and UV absorption makes the PSB-HEC3 hydrogel a versatile material for a variety of applications, including advanced health diagnostics and environmental monitoring.

### 3.2 Mechanical Properties of the Fabricated Hydrogels

To evaluate the mechanical properties of the hydrogel, rheological measurements were performed to assess the viscoelastic behavior. The crossover point, where the viscous behavior (G″) dominates over the elastic behavior (G’), was observed at 93% shear strain for the PSB-HEC3 hydrogel, significantly higher than the 20% shear strain observed for the PSB hydrogel (Figure 2c). This indicates that the inclusion of HEC strengthens the hydrogel, allowing it to withstand higher strains before rupture, and enhancing its structural integrity by increasing the strain at which the gel and its cross-links break.^38,39^ Furthermore, in the frequency sweep measurements, the elastic modulus (G′) was found to dominate over the viscous modulus (G″), confirming the solid-like, elastic nature of both gels (Figure 2d). Both G′ and G″ were higher for the PSB-HEC3 hydrogel compared to the PSB hydrogel, indicating that the incorporation of HEC enhances the overall mechanical properties of the hydrogel, providing superior strength and stability.^40^ These findings show that the HEC improves the mechanical performance of the hydrogel, making it suitable for applications requiring flexible yet durable materials.

Tensile testing was performed to further evaluate the mechanical properties of the hydrogel (Figure 3.) The tensile stress–strain curves for PSB, PSB-HEC1, PSB-HEC3, and PSB-HEC5 hydrogels are shown in Figure 3a, while the corresponding toughness and elastic moduli are shown in Figure 3b. The mechanical strength of the hydrogels is significantly influenced by the amount of HEC incorporated. The mechanical strength and elasticity of the hydrogels are significantly influenced by the amount of HEC incorporated. Compared to the neat PSB hydrogel, PSB-HEC1 and PSB-HEC3 exhibit substantial improvements in tensile strength and elastic modulus, while elongation at break remains relatively unchanged, indicating that low-to-intermediate HEC content reinforces the hydrogel network without compromising flexibility. In contrast, PSB-HEC5 shows a slight decrease in tensile strength and modulus, which may result from excessive HEC creating a denser but more heterogeneous network, leading to local stress concentrations and reduced load-bearing efficiency. On the other side, compared to the PSB hydrogel, the PSB-HEC3 hydrogel exhibits substantial increases in tensile strength (from 18 to 54 kPa), strain (from 109 to 268%), and toughness (from 1147 to 7728 kJ/m^3^). The elastic modulus remains nearly constant, increasing only slightly from 444 to 457 kPa. These enhancements can be attributed to the HEC providing rigidity to the hydrogel, while the cross-linked PSB networks contribute flexibility and elasticity through a combination of chemical cross-linking and physical interactions, including electrostatic forces and hydrogen bonding. It has also been demonstrated that lithium ions in the polymer network can help reduce local stress within the gel and balance the forces during the stretching process.^41^

**Figure 3:**
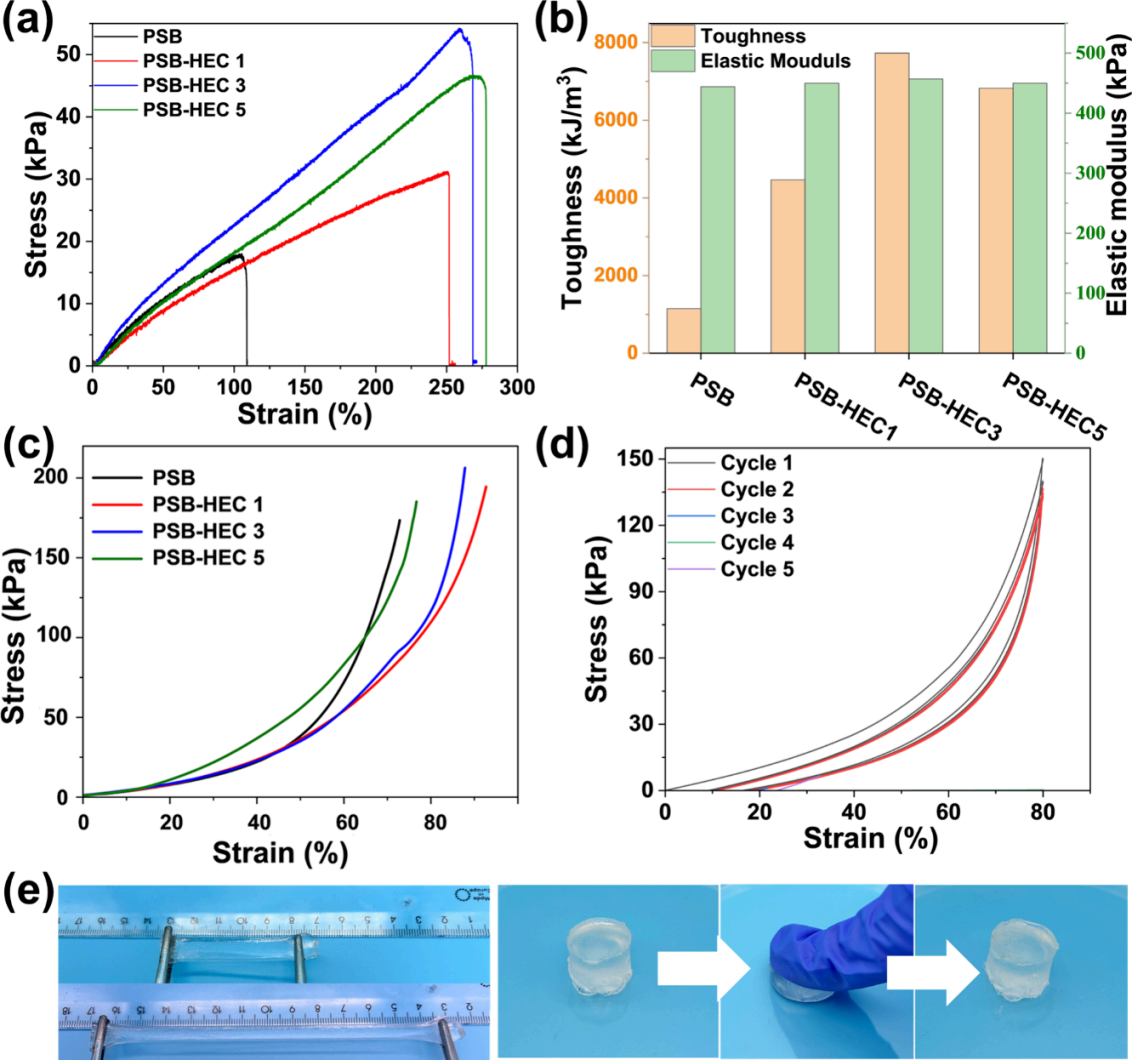
(a) Tensile stress–strain curves of PSB, PSB-HEC1, PSB-HEC3, and PSB-HEC5 hydrogels and (b) the corresponding toughness and modulus. (c) Compressive stress–strain curves, (d) cyclic compressed loading and unloading curves of PSB-HEC3 hydrogel at 80% strain, and (e) photographs of PSB-HEC3 hydrogel subjected to stretching and compression by hand.

Compression tests were also performed to evaluate the hydrogel’s response to compressive forces. The resulting compressive stress–strain curves are presented in Figure 3c. Notably, the PSB-HEC3 and PSB-HEC5 hydrogels demonstrated remarkable compressive resilience, with both able to withstand up to 80% strain without rupture. This high level of compressive deformability indicates the excellent structural integrity and robustness of these hydrogels under compressive forces, which is crucial for applications involving dynamic loading and compression. The superior performance of the PSB-HEC3 and PSB-HEC5 hydrogels in compression further emphasizes the positive impact of HEC reinforcement, enhancing the ability of the hydrogel to maintain its structural stability even under significant deformation.

Cyclic compressive tests were performed to evaluate the durability of the PSB-HEC3 hydrogel under repetitive stress, and the results are shown in Figure 3d. The PSB-HEC3 hydrogel demonstrated excellent recoverability and minimal degradation in compressive behavior even after multiple compression cycles, confirming its suitability for applications subjected to long-term cyclic loads. The mechanical properties of the hydrogels are demonstrated in photographs of the samples subjected to stretching and compression by hand in Figure 3e, offering a visual representation of their strength, stretchability, and compressibility.

### 3.3 Self-Adhesive Properties of the PSB-HEC Hydrogels

Wearable strain sensors must possess self-adhesive properties to ensure long-term functionality.^42^ To demonstrate the self-adhesive capabilities of the PSB-HEC3 hydrogel, it was applied between the human body and various objects, such as a glass vial, metallic clips, a polypropylene (PP) cap from a standard laboratory vial, and wood (Figure 4a). The hydrogel adhered firmly to both sides of each object, without the need for additional adhesives. Adhesion to PP, a chemically inert and nonpolar polymer, primarily arises from physical interactions such as van der Waals forces and limited dipole interactions, which are generally weaker than the hydrogen bonding and ion–dipole interactions observed with more polar substrates like glass and metals. The adhesive properties of the PSB-HEC hydrogels are attributed to the zwitterionic and hydroxyl groups present in the hydrogels. Zwitterionic polymers exhibit neutral net charges and high dipole moments due to the presence of cationic quaternary ammonium groups and anionic sulfonate groups within the same monomeric unit. The zwitterions can interact with other charged or polar groups via ion-dipole and dipole–dipole interactions. In addition, the hydroxyl groups enable hydrogen bonding with other substrates.^43^ The bonding mechanisms between the hydrogel and different substrates, including hydrogen bonds, metal-ion coordination, and dipole–dipole interactions,^44,45^ which are illustrated in Figure 4b. This self-adhesion is particularly advantageous for applications in wearable devices and medical applications, where a reliable and skin-friendly adhesion is essential for long-term use. The image clearly illustrates the capability of the hydrogel to conform to the skin, offering both comfort and stability.

**Figure 4:**
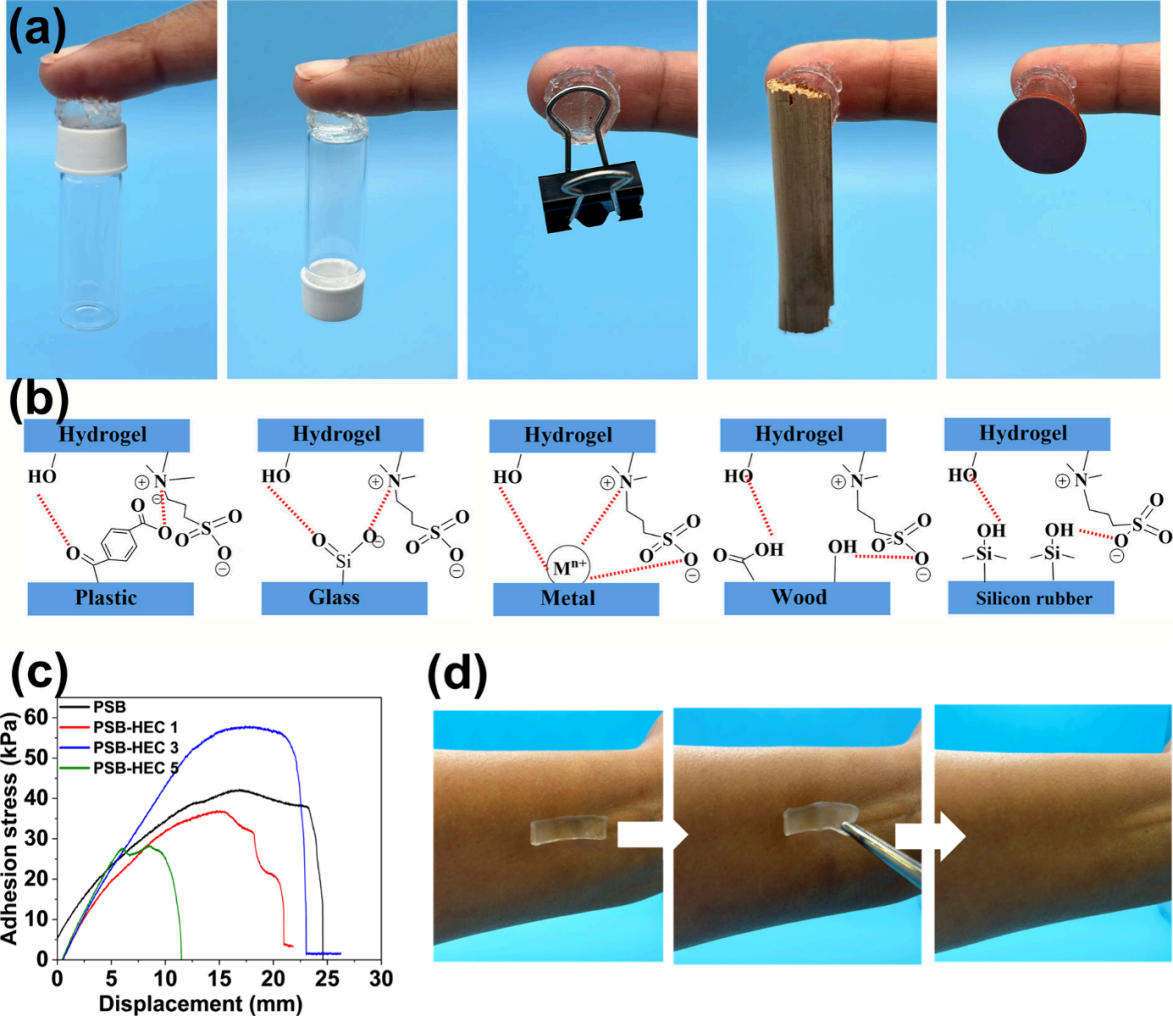
(a) Photographs showing the adhesion of the hydrogel to different surfaces, including plastic, glass, metal, wood, and silicone rubber; (b) proposed adhesion interactions of PSB-HEC hydrogels with various substrates; (c) adhesion strength of different hydrogels on corrugated paper substrates; (d) peeling off from the skin without leaving any residue.

The adhesive strength of hydrogels with varying HEC contents on paper surfaces was measured to quantitatively evaluate their adhesive properties. As shown in Figure 4c, the adhesion stress of the hydrogels reached a maximum of 40 kPa for PSB and 57 kPa for PSB-HEC3. The adhesion strength of PSB-HEC3 was slightly higher than that of the PSB-HEC1 and PSB-HEC5 hydrogels. This enhanced adhesion performance is likely due to an optimal combination of surface chemistry and polymer network structure in PSB-HEC3, which promotes stronger interfacial interactions such as hydrogen bonding and dipole–dipole interactions, thereby improving the hydrogel’s ability to adhere to different substrates.^46,47^ The adhesion strength decreased sharply after reaching its peak, which was caused by the deformation of the hydrogel samples and their peeling off from the surface.

To showcase the self-adhesive properties of the PSB-HEC3 hydrogel, it was applied to human skin, as shown in Figure 4d. The hydrogel bonded securely to the skin without the need for extra adhesives, demonstrating its capacity to form a strong attachment. The hydrogel could be effortlessly removed from the skin, leaving no residue behind, highlighting its potential for use in applications requiring easy removal and reusability without damaging the underlying surface or leaving residues.

The self-adhesive properties of the PSB-HEC hydrogels are crucial for their performance in a variety of applications, particularly in medical and wearable devices. These properties enable the hydrogels to adhere securely to surfaces, including human skin, without the need for external adhesives, offering convenience, ease of application, and improved functionality. The ability of the PSB-HEC hydrogels to conform to irregular surfaces enhances their suitability for skin-contacting products such as wound dressings, sensors, and flexible electronics. This self-adhesion, driven by the interactions between zwitterionic groups and hydroxyl groups, contributes not only to the hydrogel’s adhesive strength but also to its flexibility and stability. Moreover, the hydrogel can be easily removed without leaving residue, adding to its practicality for dynamic environments. The combination of these properties ensures that the PSB-HEC hydrogels maintain stability, reliability, and performance, making them ideal for long-term use in various applications.

### 3.4 Ionic Conductivity and Sensing Performances of the PSB-HEC Hydrogels

A conductivity measurement of PSB-HEC hydrogels was conducted to evaluate its potential as a sensor. As shown in Figure 5a, the conductivity of PSB was about 2.0 × 10^–3^ S/m. By adding the HEC, the conductivity was decreased slightly to 1.6, 1.1, and 0.77 × 10^–3^ S/m for PSB-HEC 1, 3, and 5, respectively. This result can be attributed to the fact that a polymer network composed solely of zwitterionic chains enhances ionic conductivity by promoting the migration of both Li^+^ and Cl^–^ ions.^48^ However, when HEC is introduced into the hydrogels, it interferes with the ion migration process. It should be noted that the PSB and LiCl content is similar in all gels, while HEC was added in various amounts (1, 3, or 5 wt %). The presence of HEC created neutral domains in the gel but also increased the cross-link density, which together caused a slight reduction in the overall conductivity. Regardless, ion transport pathways formed by the PSB are present through the polymer matrix, facilitating ion separation and movement of both Li^+^ and Cl^–^ ions.^49^

**Figure 5:**
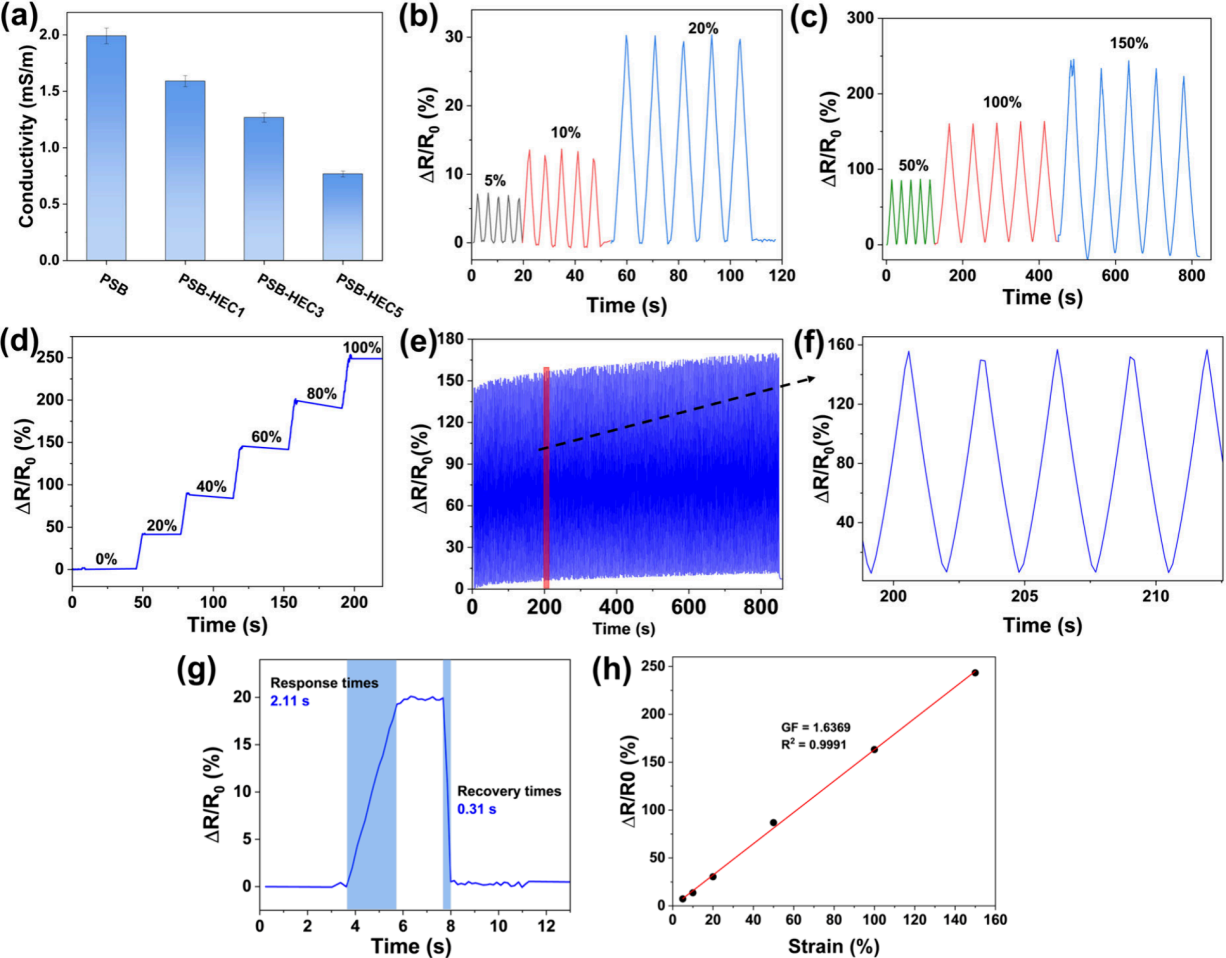
(a) The conductivity of PSB and PSB-HEC hydrogels with different HEC contents. Relative resistance changes of the PSB-HEC3 hydrogel (b) under low strains, (c) under high strains, (d) under gradient strain, and (e,f) with 100% strain for 300 cycles. (g) Response and recovery time of the PSB-HEC3 hydrogel at a strain of 10%. (h) Relative resistance changes of the PSB-HEC3 hydrogel at different strains.

The sensory performance of PSB-HEC3 was evaluated and is shown in Figure 5b–f. The relative resistance changes under low strains (5–20%) are shown in Figure 5b, indicating that the hydrogel sensor responds clearly even under small deformations. Under high strains (50–150%), the sensor also exhibits a stable response, as presented in Figure 5c, demonstrating effective operation over a wide strain range. Additionally, the hydrogel’s behavior under a gradient strain is shown in Figure 5d, confirming that the sensor response scales consistently with increasing deformation. The change in resistance of the hydrogel sensor was monitored under repetitive stress at 100% strain over 300 cycles (Figure 5e–f), demonstrating excellent stability, repeatability, and durability during continuous stretching and releasing tests. Furthermore, the response and recovery times of the PSB-HEC3 hydrogel at 10% strain are illustrated in Figure 5g and were determined to be 2.1 and 0.31 s, respectively, showing fast and reliable performance.

The sensitivity of a strain sensor is a critical parameter in determining its performance, particularly in applications that require precise monitoring of strain or deformation. The sensitivity can be evaluated using the GF factor. The gauge factor (GF) was calculated from the slope of the resistance–strain curve. For instance, Qin et al.^50^ reported a gelatin organohydrogel-based strain sensor with a GF of 1.5 within a strain range of 0–300%, while Ye et al.^51^ developed an ionogel-based strain sensor exhibiting a GF of 1.2 (0–150%) and 1.5 (150–300%). In comparison, our PSB-HEC3 sensor demonstrates a GF of 1.63 over a strain range of 0–150% (Figure 5h). Although higher GF values have been reported in some conductive hydrogels^52,53^ our value remains within the typical range for ion-conductive hydrogels., making the PSB-HEC3 hydrogel suitable for applications where precise strain measurement is essential, such as wearable sensors, health monitoring devices, and soft robotics, where accurate detection of mechanical changes can directly impact functionality and user experience.

The utilization of PSB-HEC hydrogels as strain sensors was demonstrated by attaching the sensors to various human joints (fingers, elbows, knees, and wrists). The resistance of the hydrogel changed consistently and steadily with the movement of the joints, showcasing its ability to respond accurately to dynamic deformations (Figure 6a–d). The reliable response to movement shows the sensitivity and adaptability of the hydrogel to a wide range of human motions. Demonstrating that the hydrogel is suitable for applications such as wearable devices for motion tracking, biomechanical monitoring, and health diagnostics.

**Figure 6:**
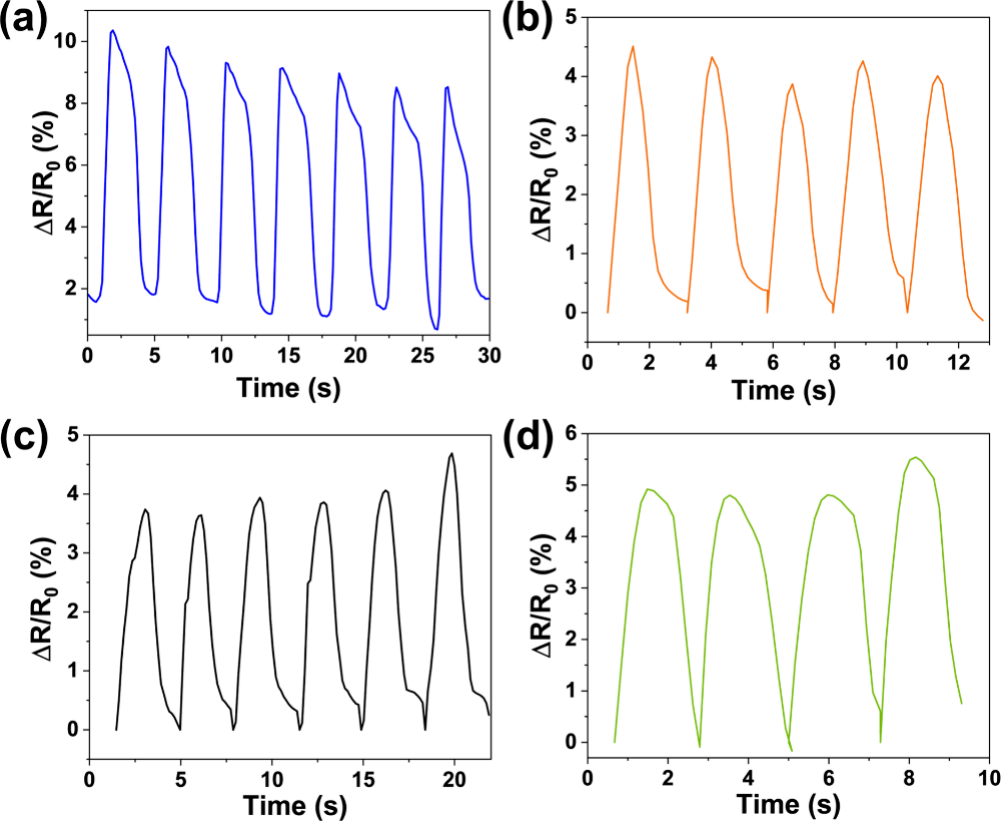
Responsiveness of the PSB-HEC3 sensor to human motion: bending of (a) a finger, (b) an elbow, (c) a knee, and (d) a wrist.

The tensile strength, sensitivity, response time, and conductivity at subzero temperatures of PSB-HEC3 hydrogel are compared with those reported in the literature in Table 1. It is worth noting that the observed performance of final hydrogels—including their mechanical strength, ionic conductivity, and adhesive behavior—is strongly influenced by the specific composition and the ratios of each polymer. The comparative analysis clearly demonstrates that the PSB-HEC3 hydrogel developed in this work achieves a well-balanced and multifunctional performance profile, setting it apart from many existing conductive hydrogels. While some reported materials exhibit higher tensile strength or gauge factors, they often lack key features such as strong adhesion, broad strain sensitivity, or antifreezing capability. These combined features underscore the significance of this work, positioning PSB-HEC3 as a versatile and durable hydrogel sensor platform.

**Table 1:** Comparison of Mechanical, Electrical, and Antifreeze Properties of PSB-HEC3 Hydrogel versus Reported Conductive Hydrogels

Hydrogel material	Tensile strength (kPa)	GF	Response time (s)	Conductivity at subzero temperature	Adhesion strength (kPa)	Reference
PSB-HEC3 (This work)	54	1.63	2.1	Yes (with LiCl)	57 (paper)	This work
P(AM-co-AMPS)/CNF/ZnSO_4_ ^a^	54.66	1.06	0.76	No	∼27.82 (glass)	^54^
PVA/TA/PAM^b^	80	0.6	-	Yes (with LiCl)	14.12 (paper)	^55^
Poly(SBMA-co-HEAA)^c^	∼45	-	-	Yes (with LiCl)	∼13 (filter paper)	^56^
PAAm-PAAc-CMC^d^	730	2.62	0.08	-	16.7	^57^
P(AA-SMA-SBMA)^e^	50–219	-	-	No	-	^39^
P(SBMA-MMA)^f^	54 kPa to 6.7 MPa	0.37	-	-	-	^58^

a Poly (acrylamide-*co*-2-acrylamide-2-methylpropane sulfonic acid)/cellulose nanofibers/ZnSO_4_.

b Poly­(vinyl alcohol)/tannic acid/polyacrylamide,

c Poly­[2-(methacryloyloxy)]­ethyldimethyl-(3-sulfopropyl)­ammonium hydroxide copolymerized with *N*-(2-hydroxyethyl)­acrylamide.

d Polyacrylamide–poly acrylic acid–carboxymethyl cellulose.

e Poly­(acrylic acid–octadecyl methacrylate–sulfobetaine methacrylate).

f Poly­(sulfobetaine methacrylate–methyl methacrylate).

### 3.5 Antifreezing Properties

Traditional hydrogels are prone to freezing at 0 °C. When water freezes, the hydrogel loses its structural integrity and mechanical properties. Freezing can limit the use of these hydrogels in low-temperature environments, as it compromises their functionality and performance. The antifreezing properties of the hydrogel were investigated by DSC. A thermogram ranging from −50 to 50 °C is shown in in Figure 7a. No exothermic or endothermic peaks can be observed in the thermogram, suggesting that the hydrogel does not freeze within the tested temperature range. It should be noted that the water content of the hydrogel was around 25% (Figure 7b). The presence of LiCl lowers the freezing point of water within the hydrogel via colligative effects, where dissolved LiCl ions disrupt the formation of ice crystals by interfering with the hydrogen bonding network of water molecules. This ion-induced freezing point depression prevents ice nucleation and growth even at subzero temperatures, thereby maintaining the hydrogel’s flexibility and structural integrity.^27,31,59^ The absence of phase transitions in the DSC trace confirms the ability of the hydrogel to maintain its structural and functional properties at low temperatures, making it suitable for use in cold environments where traditional hydrogels might fail.

**Figure 7:**
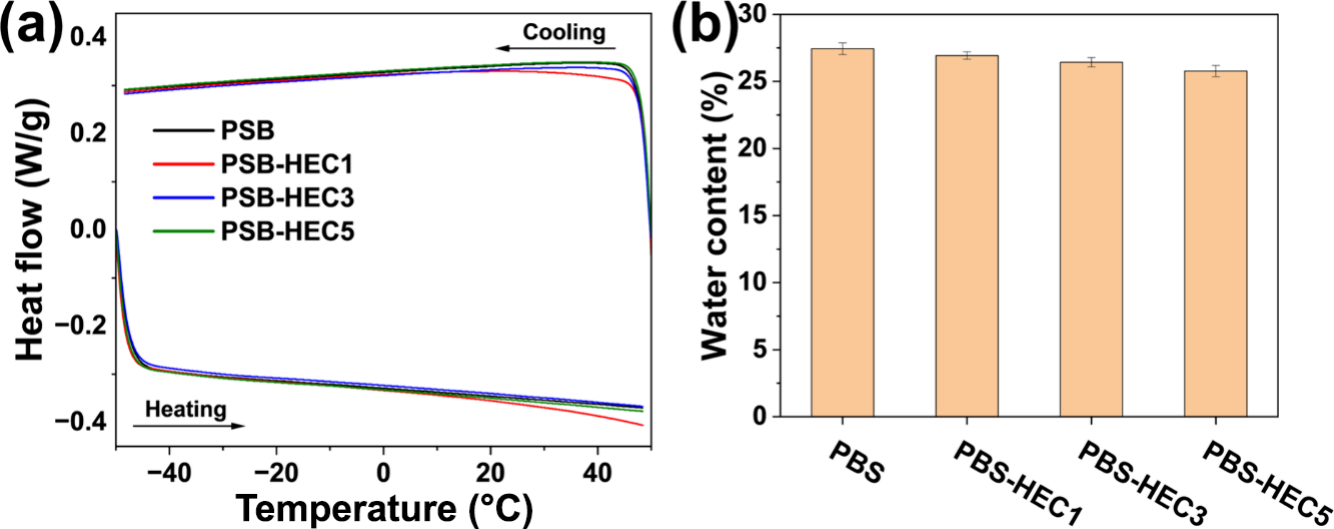
(a) DSC traces of PSB and PSB-HEC hydrogels and (b) the water content of PSB and PSB-HEC hydrogels.

## 4 Conclusions

We have developed a polyzwitterion-grafted hydroxyethylcellulose (PSB-HEC) hydrogel that demonstrates excellent mechanical, electrical, and adhesive properties, making it highly suitable for wearable sensor applications. By grafting HEC with polyzwitterions and cross-linking the system, the hydrogel retained its inherent flexibility and biocompatibility while showing ionic conductivity and mechanical strength. Importantly, the hydrogel’s antifreezing properties, evidenced by DSC analysis and maintained ionic conductivity at subzero temperatures, enable reliable sensor functionality under freezing conditions. The optimized composition of the hydrogel, PSB-HEC3, showed exceptional mechanical performance, with a tensile strength of 54 kPa, elongation at break of 268%, and an impressive toughness of 7728 kJ/m^3^—significant improvements over the nongrafted PSB hydrogel, which exhibited only 18 kPa tensile strength and 109% elongation. The enhanced flexibility, toughness, and stability of the hydrogel can be attributed to the hydrogen bonding, chemical cross-linking between zwitterionic and hydroxyl groups, as well as the integration of LiCl. The PSB-HEC3 hydrogel exhibited high adhesion strength of 57 kPa on various substrates, ensuring reliable attachment to skin, plastic, wood, and metal. The presence of the zwitterionic groups enabled strong dipole interactions, while the hydroxyl groups facilitated hydrogen bonding, leading to improved adhesion properties. The PSB-HEC3 hydrogel demonstrated excellent sensing performance across a wide range of strain levels. GF reached a notable value of 1.63, while the hydrogel sensor exhibited a fast response time of 2.1 s and a recovery time of 0.31 s. The hydrogel maintained stable performance indicated by changein resistance during 300 cycles of repeated stretching and releasing at 100% strain, showing the durability and reliability the hydrogel in continuous monitoring of human motion. The sensor effectively detected both low (5%–20%) and high (50%–150%) strain, confirming its adaptability for wearable dynamic applications. Together with the demonstrated ionic conductivity retention at subzero temperatures, the antifreezing capability expands the hydrogel’s potential use in wearable devices designed for healthcare monitoring, soft robotics, and human-machine interfaces in cold or harsh environments.

## Data Availability

Data will be made available on request.
